# Bacterial Surface Detachment during Nebulization with Contaminated Reusable Home Nebulizers

**DOI:** 10.1128/spectrum.02535-21

**Published:** 2022-02-02

**Authors:** Jamie C. Harris, Melanie S. Collins, Pamela H. Huang, Craig M. Schramm, Thomas Nero, Jing Yan, Thomas S. Murray

**Affiliations:** a Connecticut Children’s Medical Center, Division of Pediatric Pulmonology, Hartford, Connecticut, USA; b Yale School of Medicine, Department of Pediatrics, Infectious Diseases and Global Health, New Haven, Connecticut, USA; c Yale University, Department of Molecular, Cellular and Developmental Biology, New Haven, Connecticut, USA; University of North Dakota

**Keywords:** nebulizer contamination, bacterial aerosolization, surface rewetting

## Abstract

Patients with chronic respiratory diseases use home nebulizers that are often contaminated with pathogenic microbes to deliver aerosolized medications. The conditions under which these microbes leave the surface as bioaerosols during nebulization are not well characterized. The objectives of this study were to (i) determine whether different pathogens detach and disperse from the nebulizer surface during aerosolization and (ii) measure the effects of relative humidity and drying times on bacterial surface detachment and aerosolization. Bacteria were cultured from bioaerosols after Pari LC Plus albuterol nebulization using two different sources, as follows: (i) previously used nebulizers donated by anonymous patients with cystic fibrosis (CF) and (ii) nebulizers inoculated with bacteria isolated from the lungs of CF patients. Fractionated bioaerosols were collected with a Next-Generation Impactor. For a subset of bacteria, surface adherence during rewetting was measured with fluorescence microscopy. Bacteria dispersed from the surface of used CF patient nebulizers during albuterol nebulization. Eighty percent (16/20) of clinical isolates inoculated on the nebulizer in the laboratory formed bioaerosols. Detachment from the plastic surface into the chamber solution predicted bioaerosol production. Increased relative humidity and decreased drying times after inoculation favored bacterial dispersion on aerosols during nebulized therapy. Pathogenic bacteria contaminating nebulizer surfaces detached from the surface as bioaerosols during nebulized therapies, especially under environmental conditions when contaminated nebulizers were dried or stored at high relative humidity. This finding emphasizes the need for appropriate nebulizer cleaning, disinfection, and complete drying during storage and informs environmental conditions that favor bacterial surface detachment during nebulization.

**IMPORTANCE** Studies from around the world have demonstrated that many patients use contaminated nebulizers to deliver medication into their lungs. While it is known that using contaminated medications in a nebulizer can lead to a lung infection, whether bacteria on the surface of a contaminated nebulizer detach as bioaerosols capable of reaching the lung has not been studied. This work demonstrates that a subset of clinical bacteria enter solution from the surface during nebulization and are aerosolized. Environmental conditions of high relative humidity during storage favor dispersion from the surface. We also provide results of an *in vitro* assay conducted to monitor bacterial surface detachment during multiple cycles of rewetting that correlate with the results of nebulizer/bacterial surface interactions. These studies demonstrate for the first time that pathogenic bacteria on the nebulizer surface pose a risk of bacterial inhalation to patients who use contaminated nebulizers.

## INTRODUCTION

Inhaled medications delivered via home nebulizers are a critical part of health maintenance for people with chronic respiratory diseases, such as asthma, chronic obstructive pulmonary disease (COPD), and cystic fibrosis (CF). In fact, patients with CF develop chronic respiratory infections that contribute to morbidity and mortality (1–4). Unfortunately, the home nebulizers are frequently contaminated with both oral flora and pathogenic microbes (e.g., Staphylococcus aureus and Pseudomonas aeruginosa) that if aerosolized may reach the respiratory tract (5–8). While organizations such as The Cystic Fibrosis Foundation (CFF) have guidance for the proper cleaning and disinfection of home nebulizers after each use to prevent contamination (9), families often have difficulty adhering to these recommendations due to time constraints (5). Another contributing factor to nebulizer contamination is the final drying step (10, 11). While complete drying decreases the viability of P. aeruginosa, active drying with a paper towel can lead to bacterial reintroduction (10, 11). In one hospital, the use of improperly dried nebulizers contaminated with P. aeruginosa resulted in respiratory infections in CF patients (12).

**FIG 1:**
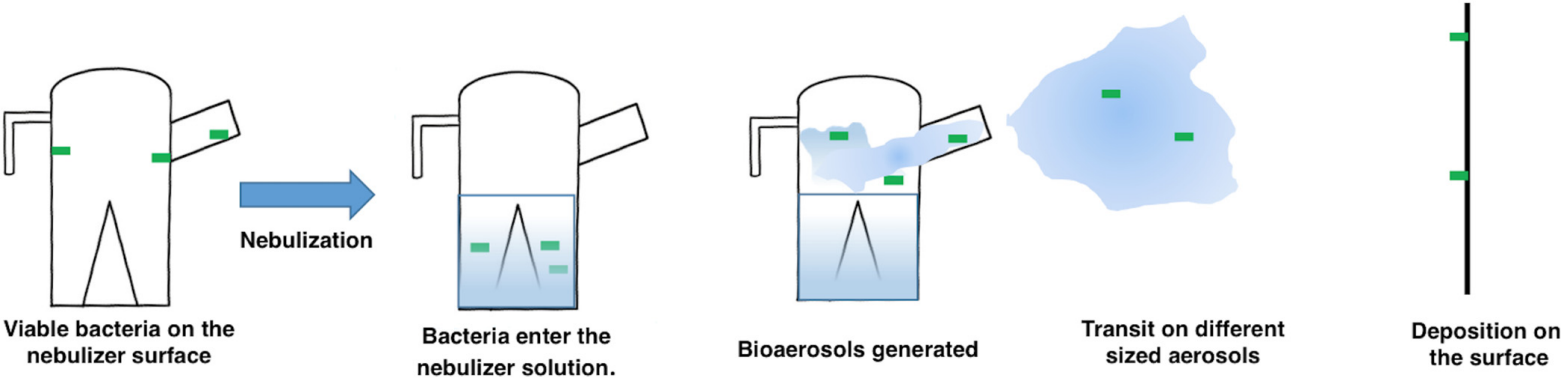
Potential exposure to the patient when using a contaminated nebulizer. Bacteria viable on the nebulizer surface must be detached and aerosolized during medication use, survive as a bioaerosol, and finally retain viability after the microbes are deposited in the respiratory tract.

Several elements likely determine whether bacteria will leave the plastic nebulizer surface during the active production of respiratory aerosols. (Fig. 1). Microbes dry by nonuniform evaporation at the air-liquid interface, resulting in droplets drying in a coffee-ring pattern (13). Viability during coffee-ring formation is affected by the immediate environmental conditions, such as temperature, relative humidity (RH), availability of water and nutrients, and salt concentrations (14–16). Next, the microbes must detach from the nebulizer surface, enter solution, and subsequently form bioaerosols during the surface rewetting that occurs while aerosolizing a liquid solution. Multiple factors that influence this process include but are not limited to the material and charge of the surface as well as bacterial surface associated molecules (e.g., exopolysaccharide, flagella, and pili) (17–22). Bacteria traveling on aerosolized medication droplets must then remain viable until deposited on a new surface. This process is also dependent on the water content, temperature, and RH of the air and the bacterial properties in the bioaerosol (11, 16, 23). In the case of nebulized therapy, the bioaerosol only has to travel a short distance from the nebulizer chamber through the neck and mouthpiece into the airway. Finally, the dispersed microbes must survive impact with the surface of the airway.

We hypothesized that using contaminated home nebulizers expose the lungs to aerosolized pathogenic bacteria. To better understand this risk and the environmental conditions that determine bacterial surface detachment during aerosol production from liquids, we studied the detachment and aerosolization of bacteria from contaminated nebulizer surfaces during the nebulization of albuterol, a bronchodilator used commonly by asthmatics. Furthermore, we characterized the factors during nebulizer care and storage that influence bacterial surface interactions and aerosolization.

## RESULTS

### Bacteria from contaminated patient nebulizers are recovered from aerosols.

**FIG 2:**
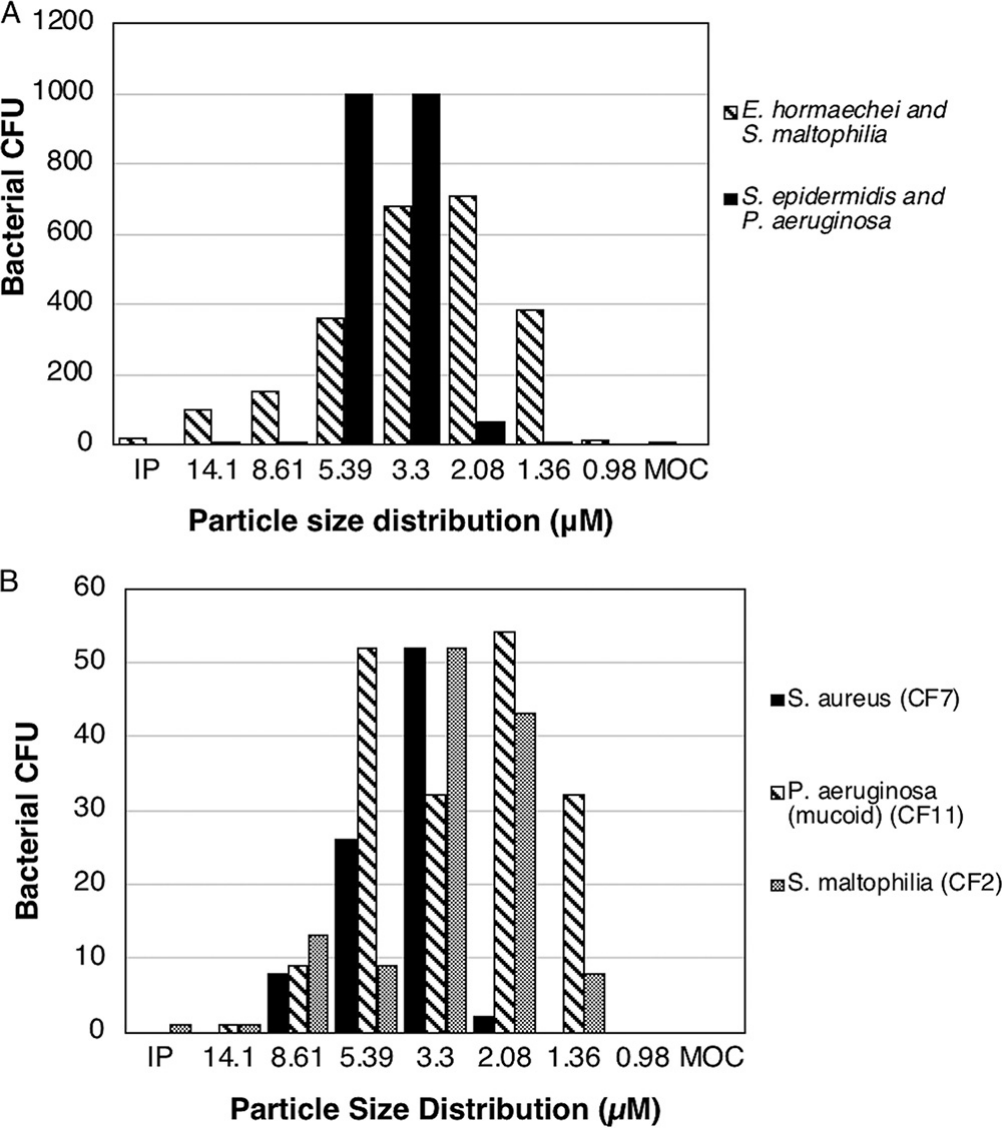
Nebulizers contaminated on the surface in the laboratory with *S. aureus, P. aeruginosa*, or *S. maltophilia* dispersed viable bacteria during albuterol nebulization. (A) NGI collecting cups containing aerosols of different sizes were swabbed for bacteria after albuterol nebulization with two different used patient PARI LC Plus nebulizers. (B) Pathogenic CF respiratory isolates were aerosolized into the conductive (>5 μM) and respirable (<5 μM) fractions. Bacteria from contaminated nebulizers dispersed on bioaerosols. The individual representative isolates were dried on the nebulizer surface for 2 h at ambient RH (54% to 65%). MOC, micro-orifice.

There are little data on whether bacteria contaminating the surface of nebulizers are aerosolized during nebulization of sterile liquid. As a proof of concept, we first examined used patient nebulizers to determine whether bacteria from patients leave the nebulizer surface as bioaerosols. Thirty percent (3/10) of previously used PARI LC Plus nebulizers provided by children with CF were colonized with bacteria that dispersed during albuterol nebulization on different aerosolized particle sizes (Fig. 2A). One nebulizer was contaminated with Bacillus cereus (4,073 recovered aerosolized CFU), one with Enterobacter hormaechei and Stenotrophomonas maltophilia (2,406 recovered aerosolized CFU), and another with Staphylococcus epidermidis and a Pseudomonas sp. (2,072 recovered aerosolized CFU) identified as most consistent with P. aeruginosa (Fig. 2A and data not shown). All organisms traveled on respirable particles of <5 μm with the potential to reach the lower airway (Fig. 2A and data not shown).

### CF respiratory isolates are aerosolized on particles of different sizes.

Next, we aerosolized albuterol with Pari LC Plus nebulizers contaminated in the laboratory with bacterial pathogens and collected the dispersed aerosols in the Next Generation Pharmaceutical Impactor (NGI). Importantly, 80% (16/20) of clinical isolates (CF#) produced bioaerosols capable of reaching the airway, including bacteria from each studied genus (Table 1; Fig. 2B; see Table S1 in the supplemental material). Thirty-six percent (21/58) of the albuterol nebulization experiments resulted in the recovery of pathogenic bacteria from bioaerosols (Table 1; Fig. 2B; Table S1). We did not detect a difference when comparing dispersion among different bacterial species (analysis of variance [ANOVA], F(4,28) = 1.11, *P* = 0.37) or when comparing different inoculum amounts (simple regression, R^2^ = 0.044, F(1,31) = 1.42, *P* = 0.24). However, the recovery of organisms detached from the surface into the nebulizer solution was a key factor in bioaerosol production, such that dispersion occurred in 20/39 (51.3%) trials with bacteria present in solution compared with 1/19 (5.3%) trials where the bacteria was recovered only from the nebulizer surface and NGI but not in solution (odds ratio of dispersion once in solution of 9.74; 95% confidence interval [CI], 1.21 to 78.14; *P* = 0.032).

**TABLE 1:** Bioaerosols are produced after aerosolizing albuterol with surface-contaminated nebulizers

CF respiratory isolate	No. (%) of strains that produce viable bioaerosols	Median CFU (range) recovered of:		
		Total	Aerosol particle size 3.3–0.98 μM	Particle size of 14.1–5.39 μM
S. aureus (*n* = 6)	4 (66)	9 (0–1,223)	4 (0–913)	3 (0–310)
P. aeruginosa, nonmucoid (*n* = 4)	3 (75)	0.5 (0–393)	0 (0–301)	0 (0–92)
P. aeruginosa, mucoid (*n* = 4)	4 (100)	3 (0–717)	2 (0–212)	1 (0–505)
S. maltophilia, (*n* = 3)	3 (100)	4.5 (0–973)	3.5 (0–715)	0 (0–258)
*Burkholderia* sp., (*n* = 3)	2 (66)	1 (0–203)	0.5 (0–155)	0.5 (0–48)
All isolates (*n* = 20)	16 (80)	3 (0–1,223)	1 (0–913)	1 (0–505)

### The effect of ambient RH and extended drying on bacterial viability, and aerosolization.

**FIG 3:**
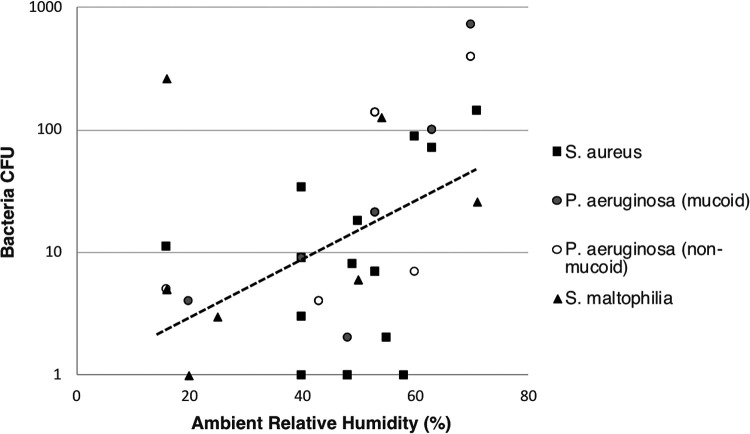
Nebulizer drying at higher relative humidity correlates with increased bacterial dispersion. Each marker displays the results from an individual experiment involving a single isolate contaminating a nebulizer. Dotted line depicts regression of all organisms. Fifty-eight experiments involving 20 strains are shown.

We noticed a positive correlation between increased ambient RH during nebulizer drying and increased recovery of bacteria in the NGI from dispersed aerosols (simple regression, R^2^ = 0.172, F(1,31) = 6.45, *P* = 0.02) (Fig. 3). Although the nebulizer surface was visibly dry on inspection at 2 h after inoculation, we hypothesized that isolates had increased dispersion into the NGI at high RH due to incomplete drying, facilitating entry into the aerosolized liquid solution. To understand this relationship for individual strains, we repeated experiments after drying bacteria from an overnight culture at either low (≤25%) or high (>60%) RH. Nebulizer drying for 2 h at low RH reduced aerosolization compared with drying at high RH (mean, 0.85 ± 0.48 log CFU versus 2.05 ± 0.29 log CFU; *P* = 0.039) (see Table S2 in the supplemental material). Furthermore, when data from original experiments at ambient RH were pooled with experiments that controlled for humidity (Fig. 3 combined with Table S2), bacterial dispersion remained associated with increased RH (simple regression, R^2^ = 0.231, F(1,47) = 14.13, *P* = 0.0005).

Next, we hypothesized that extended drying would reduce dispersion at increased RH. Comparing the same inoculum dried for either 2 or 24 h at higher RH, we found that while extended drying did not eliminate bacterial dispersion, the trend was that fewer bacteria were recovered from bioaerosols after 24 h of drying (Mann-Whitney U-test, *n* = 6 per group, *P* = 0.055) (see Table S3 in the supplemental material).

### Surface retention during rewetting is inversely proportional to dispersion during nebulization.

**FIG 4:**
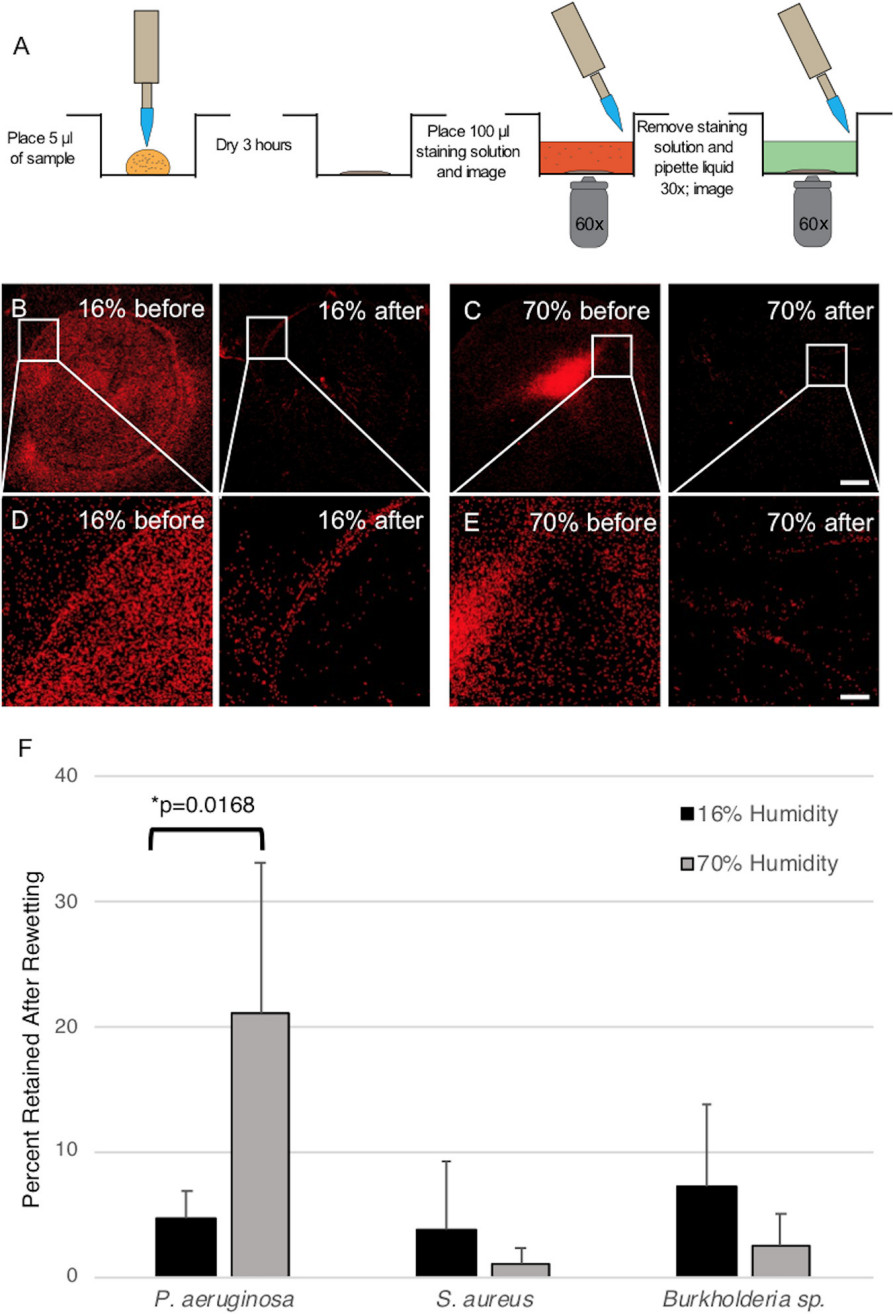
Bacterial surface retention after rewetting visualized by fluorescence microscopy. (A) Schematic of the assay to measure bacterial surface retention on glass after washing 30 times with water to mimic the repeated wetting/rewetting process during nebulization. (B to E) Representative coffee-ring structure formed by drying a bacterial suspension of *Burkholderia* sp. (CF50) at 70% (B and D) or 16% (C and E) relative humidity (RH). Images both before and after the repeated wetting step are shown. (D and E) Magnified views of the same area of the coffee-ring formation seen in B and C, respectively. (F) Percentage of cells retained on the surface after repeated wetting for each strain at 16% or 70% RH, respectively, for P. aeruginosa (CF23), S. aureus (CF7), and *Burkholderia* sp. (CF50). An image dilation operation was performed in Nikon Element software to ensure proper presentation of the bacteria on a large-scale view. Data are shown as the mean ± SD (*n *> 3; *, *P < *0.05). Scale bar: 500 μm in C and 100 μm in E.

We further examined surface retention of bacteria with microscopy in a rewetting model. To mimic the physical environment during nebulizer operation, we designed a procedure to quantify the tendency of bacterial cells to adhere to and detach from surfaces after repeat washing on a glass surface suitable for imaging (Fig. 4A). Dried bacterial solutions displayed a coffee-ring pattern (Fig. 4B to E), which is characteristic for particulate suspensions (13). After the vigorous washing step, a significant portion of cells (60% to 95%) left the surface and entered into the solution (Fig. 4F). These cells likely correspond to those that entered the nebulizing solution and were more likely to be carried by aerosolized droplets and deposited into the NGI.

Importantly, the measured retention index for each isolate corresponded well with surface retention on the nebulizer. Isolates (e.g., CF7 S. aureus, and CF50 *Burkholderia* sp.) that showed lower retention on the surface (<10%) were found in solution after albuterol nebulization. Although not statistically significant, we also observed that a higher fraction of cells detached from the surface at high humidity (70%) than that at low humidity (16%) (CF7 S. aureus, *P* = 0.4452; CF50 *Burkholderia* sp., *P* = 0.2265). Interestingly, CF23 (a mucoid P. aeruginosa) displayed an opposite pattern with more bacteria in solution at low RH (Table S2), and this finding was confirmed in the rewetting assays (*P* = 0.0168) (Fig. 4F).

## DISCUSSION

Nebulizer contamination with pathogenic organisms is a persistent, well-characterized problem for patients with chronic lung disease who routinely use home nebulizers. It is known that nebulization of a contaminated solution can be harmful (24). However, the risk of infection for patients who use a contaminated nebulizer to deliver a previously sterile medication is not known (5, 25). This information is important because the use of contaminated nebulizers is common while the rigorous manufacturing standards reduce the risk of using a contaminated medication. This study demonstrates that bacterial pathogens on used nebulizers from patients can detach from the nebulizer surface during delivery of inhaled medications on differently sized bioaerosols that are capable of dispersing throughout the respiratory tract.

Although we found an association between higher RH and bioaerosol production, there was variability between strains from the same genus with some strains always remaining surface attached and others consistently detaching and being recovered from aerosols in the NGI. This finding implies that regardless of the environmental conditions, there can be intrinsic bacterial properties that determine whether that isolate remains attached during exposure to the sheer force of nebulization. Examples of factors that may differ between isolates of the same species that are important for bacterial surface interactions include bacterial components, such as flagella in motile Gram-negative pathogens and alginate in mucoid P. aeruginosa (21, 22). We are currently evaluating the surface properties of isolates that consistently aerosolize or remain attached regardless of environmental conditions to better predict which clinical isolates may present a greater risk to the patient if recovered from the nebulizer.

The microscopy-based assay confirmed the results in the nebulizer-NGI experiment and offers a convenient approach for mechanistic studies on surface detachment into solution, which is a key initial step in forming bioaerosols. Both bacterial and abiotic surface properties, such as hydrophobicity and charge that affect coffee-ring formation, are likely to influence detachment and subsequent bioaerosol production. A biophysical approach combined with clinical data will be fruitful for predicting the risk associated with nebulizer bacterial contamination for patients who require inhaled medication.

Proper drying after nebulizer disinfection is important to prevent contamination as active drying can introduce bacteria (10). A recent study of six P. aeruginosa isolates found that complete drying prevented the recovery of viable bacteria at 24 h (11). One variable that impacts nebulizer drying is the ambient RH, with faster drying as the RH decreases. Importantly, complete drying may not be achieved even when the nebulizer looks dry to visible inspection especially in areas of high ambient RH. Drying and storing nebulizers in low humidity containers, as done with packaged foods containing desiccator packages, perhaps could be a novel, practical, and cost-effective final step after cleaning and disinfection to reduce the risk of bacterial recolonization, detachment, and aerosolization.

There are limitations to this study. (i) The used patient nebulizers were collected anonymously without clinical information, such as patient respiratory cultures, time of last use, and most recent nebulizer cleaning and disinfection. (ii) A previous study demonstrated that mucus does not alter P. aeruginosa surface viability (11). Whether mucus or oral secretions alter coffee-ring formation, bacterial surface detachment, or the properties of bioaerosols generated during nebulization is on-going work. (iii) Bacteria can be damaged during impaction with the metal collecting cup, reducing viability and underestimating the number of bacteria that survive on bioaerosols. (iv) We selected the Pari LC Plus because it is the most common nebulizer used by our patient population and albuterol because drug concentrations are measured easily to monitor nebulizer function. It is unknown if these results are applicable to other nebulizers with different design geometries and other inhaled medications.

Despite these limitations, our results demonstrate that bacterial surface attachment is disrupted during nebulization and patient isolates of pathogenic bacteria can enter the solution and disperse as bioaerosols. This finding demonstrates that the well-documented problem of nebulizer contamination poses a risk of pathogen exposure to the patient and emphasizes the need to clean and disinfect home nebulizers. Future studies will determine whether this work may be applicable to other common contaminated surfaces that undergo cycles of rewetting and drying, such as shower heads, faucets, and sink basins where contaminated aerosols can be generated and complete drying is unlikely to take place. These experiments may also augment future recommendations on how home medical equipment is stored, with considerations for low humidity drying chambers, especially during times when ambient RH is high.

## MATERIALS AND METHODS

### Sources of bacteria.

Ten used Pari LC Plus nebulizers (Midlothian, VA) were received from children during a routine visit to the Pediatric CF clinic at Yale New Haven Children’s Hospital. No information about the patient or nebulizer (e.g., last date of use or disinfection) was collected. The nebulizers were cultured for bacteria after aerosolization of albuterol, a bronchodilator commonly used for asthma (see below), and recovered bacteria were identified by the Yale New Haven Clinical Microbiology laboratory using matrix-assisted laser desorption ionization–time of flight mass spectrometry (MALDI-TOF/MS) with the Vitek (bioMérieux) *in vitro* diagnostic database. Additional strains were chosen sequentially from a library of 200 CF respiratory isolates (annotated CF1-CF200) collected from the Yale New Haven Hospital Clinical Microbiology laboratory (institutional review board [IRB] waiver 2000024319). Of these isolates, 20 that survived ≥24 h on the nebulizer surface were selected for further study (Table 1). Mucoid P. aeruginosa was classified separately because of this distinct phenotype (22).

### Recovery of bacteria from used patient and laboratory contaminated nebulizers.

Used patient nebulizers were collected from the CF clinic in individual biohazard bags. Sterile cotton swabs were dipped in lysogeny broth (LB) and wiped over 2- by 2-cm areas of the mouthpiece, the neck, and the inner chamber, and the swabs were plated onto LB agar (26). The plates were incubated at 37°C overnight and CFU recorded. For laboratory-contaminated nebulizers, respiratory isolates (Table 1) were inoculated to the Pari LC Plus nebulizers in the same three locations as above with 10 μL of a 1:1,000 dilution from an overnight culture grown in LB at 37°C that was quantified by serial dilution. Only bacterial strains where viable bacteria were recovered from the nebulizer surface and/or albuterol solution post-aerosolization were subsequently analyzed, excluding bacterial strains that did not survive drying and nebulization.

### Measurement of particle size and bacterial aerosol production.

A refrigerated Next Generation Pharmaceutical Impactor (NGI) (MSP, Shoreline, MN) measured bacterial dispersion from the nebulizer surfaces on different aerosolized particle sizes generated by a Pari LC Plus nebulizer (see Fig. S1 in the supplemental material) (27). The laboratory contaminated nebulizers were dried for 2 h, and 5 mL of albuterol 0.083% (Nephron, West Columbia, SC) was nebulized through the NGI for 7 min, a time chosen because it is just prior to the sputter (27). Albuterol was chosen because it is commonly used by patients and its measured concentration from different droplet sizes determines the nebulizer function by calculating the median mass airway diameter (Supplemental Methods). After albuterol nebulization, the following areas were cultured for bacteria: (i) inoculation sites on the head, neck, and chamber of the nebulizer; (ii) 100 μL of the estimated 3 mL of albuterol solution remaining in the chamber (limit of detection approximately 30 CFU), and (iii) The NGI collecting cups containing aerosols of various sizes. CFU were counted on LB agar plates after 24 to 48 h of incubation at 37°C. Bacterial dispersion from the nebulizer surface to viable deposition in the NGI was conservatively defined as recovering ≥5 CFU from the NGI to account for possible contamination from the local environment and air. All clinical strains were tested with a minimum of two independent experiments. If two trials gave discrepant results, a third trial was performed.

### Effect of ambient RH on bacterial dispersion.

To determine the effect of ambient RH on bacterial dispersion, we repeated experiments after nebulizer drying under conditions of high RH (60% to 70% with water bubbled into a closed container with forced air) and low RH (≤25% with desiccant gypsum chips [Drierite, Xenia, OH] in a closed chamber). The drying time was also extended from 2 h to 24 h to assess the impact of prolonged drying on bacterial surface dispersion. Temperature and RH were recorded with a combination thermometer and hygrometer (Acurite, Lake Geneva, WI).

### Bacterial surface interactions during rewetting.

The surface interaction of representative isolates that either remained attached to the surface or aerosolized well during nebulization were compared in a model of rewetting bacteria dried on a surface. Overnight bacterial cultures grown in LB at 37°C were normalized to an optical density at 600 nm (OD_600_) of 2.5 and subsequently diluted 1:100 in LB. Five microliters of the suspension was carefully spotted onto the middle of a glass well in a 96-well plate (Mat Tek/P96G-1.5-5-F) and dried for 3 hours at either 16% or 70% RH. Once dried, the bacteria were loaded with the membrane dye FM4-64 (ThermoFisher; T3166) and the cytoplasm dye CytoX (ThermoFisher; S7020) 15 minutes prior to viewing. Imaging was performed with a spinning disk confocal microscope (Nikon Ti2-E connected to Yokogawa W1), and the images were captured with a sCMOS camera (Photometrics Prime BSI; Teledyne Photometrics, Tuscon, AZ) (see Supplemental Methods for more detail). The chamber was then wetted repeatedly with water followed by removal 30 times, and the same field was captured after the vigorous wash. The procedure was subsequently repeated for each sample well. Image analysis was performed with the built-in functions in the Nikon Element software.

### Statistical analysis.

A *P* value of less than 0.05 was considered statistically significant for all tests. Chi-square analysis for nominal variables and analysis of variance or covariance (ANOVA or ANCOVA, respectively) for continuous variables were used to explore relationships of aerosolization of bacteria to bacterial species, inoculum size, presence of bacteria entering solution during nebulization, and RH. Dispersion with RH was further assessed with a simple regression. Statistical significance for rewetting experiments was determined by performing an unpaired *t* test by comparing each strain at 16% and 70% humidity with Prism. The distribution of normality was evaluated by Komogorov-Smirnov tests. Mann-Whitney U tests were done on nonnormal data sets. Other statistical analyses were performed using Statview analysis software (SAS Institute, Cary, NC). Results are reported as medians or means ± standard errors of the mean or as frequencies (%).
