# Supplementary material for: Bacterial Surface Detachment during Nebulization with Contaminated Reusable Home Nebulizers
Source: Microbiol Spectr. 2022 Feb 2;10(1):e02535-21. doi: 10.1128/spectrum.02535-21 (PMC8809330; doi:10.1128/spectrum.02535-21)
Supplement: SUPPLEMENTAL FILE 1 — Supplemental material. Download SPECTRUM02535-21_Supp_1_seq8.pdf, PDF file, 0.5 MB [file spectrum02535-21_supp_1_seq8.pdf]

ONLINE SUPPLEMENT FOR

# BACTERIAL SURFACE DETACHMENT DURING NEBULIZATION WITH CONTAMINATED REUSABLE HOME NEBULIZERS

Jamie C. Harris, Melanie S. Collins, Pamela Huang, Craig M. Schramm, Thomas Nero, Jing Yan, Thomas S. Murray

Supplementary Methods:

*Measurement of aerosolized particles and bacteria by the NGI:* An acrylic flow meter confirmed the Vios® air compressor flow at 12L/min for the nebulizers during albuterol aerosolization. The vacuum pulled through the NGI was measured inline to be within 5% of 15L/min [14.25-15.75L/min) during all experiments, per the manufacturer's instructions. At this flow rate the bioaerosols are fractionated by the NGI into the following sizes: 14.1$\mu$M, 8.61 $\mu$M, 5.39 $\mu$M, 3.3 $\mu$M, 2.08 $\mu$M, 1.36 $\mu$M, and 0.96 $\mu$M. The median mass aerosol diameter (MMAD) of the aerosolized particles after nebulization is a measurement of nebulizer function. The For each new Pari LC Plus® the MMAD was calculated measuring the distribution of albuterol after a 4-minute nebulization (S1-S4). This was repeated every 20-25 nebulizations to monitor changes in nebulizer function (S5, S6). MMADs ranged from 3.7 to 4.3 for the nebulizers used consistent with previously published literature (S7). Between trials, nebulizers were disinfected with 70% ethanol per the Cystic Fibrosis Foundation Infection Prevention and Control guidelines (S8). Cultures of the NGI system and collecting cups checked for sterility between experiments.

*Analysis of retained bacteria after repeated wetting.* Once the bacteria were dry on the surface, 100$\mu$l of M9 media containing the membrane dye FM4-64 (ThermoFisher, T3166) and cytoplasm dye CytoX (ThermoFisher, S7020), both at 1000x dilution from the stock, were added the well 15 minutes prior to imaging.

The diameter of the coffee ring was measured under 10x magnification using the program to assist with the capturing of a large field view at a higher magnification. The attached bacterial cells were subsequently imaged using a 60x water objective. The center of the ring was estimated using the measurements found under 10x magnification. FM-4-64 was viewed via excitation/emission 561/605 and CytoX was viewed via excitation emission 488/520. The CytoX was visible with the 10X objective to establish the size of the "coffee ring" and define the field for capture with the 60X objective, but only stains dead cells. FM-4-64 labels the membrane of *all* bacteria, but was only visualized at higher magnification. The imaging chamber was then repeatedly wetted 30 times and the same field was captured after the vigorous wash.

Image analysis was performed with the Nikon Element software by thresholding each image and measuring the binary area. The percentage of cells retained on the surface was calculated for each strain by dividing the binary area after the rewetting process by the area before the process and multiplying by 100. Statistical significance was analyzed by performing an unpaired t-test by comparing each strain at 16% and 70% humidity.

| | | | | CFU's recovered post nebulization | | | |
|---|---|---|---|---|---|---|---|
| Isolate | Bacteria | Ambient Relative Humidity (%) | Inoculum (CFUs) each nebulizer location | 100 μl albuterol solution | Bioaerosols 3.3-0.98 μM | Bioaerosols 14.1-5.39 μM | Total recovered from NGI |
| CF1 | *S. aureus* | 40 | 2460 | 2 | 6 | 3 | 9 |
| CF1 | *S. aureus* | 53 | 7800 | 6 | 4 | 3 | 7 |
| CF4 | *S. aureus* | 16 | 2220 | 59 | 0 | 11 | 11 |
| CF4 | *S. aureus* | 71 | 3320 | 37 | 26 | 119 | 143 |
| CF4 | *S. aureus* | 49 | 3850 | 15 | 7 | 1 | 8 |
| CF4 | *S. aureus* | 40 | 9500 | 0 | 1 | 2 | 3 |
| CF4 | *S. aureus* | 63 | 40500 | 66 | 59 | 12 | 71 |
| CF5 | *S. aureus* | 55 | 1504000 | 202 | 0 | 2 | 2 |
| CF5 | *S. aureus* | 58 | 2480000 | 0 | 0 | 1 | 1 |
| CF7 | *S. aureus* | 60 | 5000 | 15 | 54 | 34 | 88 |
| CF7 | *S. aureus* | 40 | 9000 | 17 | 4 | 5 | 9 |
| CF12 | *S. aureus* | 50 | 160000 | 2 | 0 | 0 | 0 |
| CF12 | *S. aureus* | 40 | 164200 | 328 | 1 | 0 | 1 |
| CF12 | *S. aureus* | 48 | 1430 | 0 | 0 | 1 | 1 |
| CF46 | *S. aureus* | 40 | 820 | 27 | 17 | 17 | 34 |
| CF46 | *S. aureus* | 50 | 12000 | 4 | 10 | 8 | 18 |
| CF8 | *P. aeruginonsa* (non-mucoid) | 16 | 1500 | 0 | 0 | 0 | 0 |
| CF8 | *P. aeruginonsa* (non-mucoid) | 53 | 4000 | 10 | 96 | 41 | 137 |
| CF8 | *P. aeruginonsa* (non-mucoid) | 20 | 782000 | 2 | 0 | 0 | 0 |
| CF8 | *P. aeruginonsa* (non-mucoid) | 16 | 183600000 | 33 | 0 | 0 | 0 |
| CF9 | *P. aeruginonsa* (non-mucoid) | 16 | 2790 | 0 | 0 | 0 | 0 |
| CF9 | *P. aeruginonsa* (non-mucoid) | 40 | 3790 | 0 | 0 | 0 | 0 |
| CF9 | *P. aeruginonsa* (non-mucoid) | 70 | 7280 | 3 | 301 | 92 | 393 |
| CF9 | *P. aeruginonsa* (non-mucoid) | 33 | 8100 | 103 | 0 | 0 | 0 |
| CF9 | *P. aeruginonsa* (non-mucoid) | 16 | 1456000 | 10 | 3 | 2 | 5 |
| CF17 | *P. aeruginonsa* (non-mucoid) | 40 | 1370 | 1 | 0 | 0 | 0 |
| CF17 | *P. aeruginonsa* (non-mucoid) | 40 | 256000 | 23 | 0 | 0 | 0 |
| CF56 | *P. aeruginonsa* (non-mucoid) | 60 | 4000 | 0 | 2 | 5 | 7 |
| CF56 | *P. aeruginonsa* (non-mucoid) | 43 | 4800 | 77 | 4 | 0 | 4 |
| CF11 | *P.aeruginosa* (mucoid) | 38 | 2000 | 1 | 0 | 0 | 0 |
| CF11 | *P.aeruginosa* (mucoid) | 37 | 2000 | 0 | 0 | 0 | 0 |
| CF11 | *P.aeruginosa* (mucoid) | 39 | 5020 | 0 | 0 | 0 | 0 |
| CF16 | *P.aeruginosa* (mucoid) | 20 | 2010 | 4 | 3 | 1 | 4 |
| CF16 | *P.aeruginosa* (mucoid) | 70 | 3130 | 67 | 212 | 505 | 717 |
| CF16 | *P.aeruginosa* (mucoid) | 63 | 3088000 | 101 | 61 | 38 | 99 |
| CF23 | *P.aeruginosa* (mucoid) | 48 | 1290 | 11 | 0 | 0 | 0 |
| CF23 | *P.aeruginosa* (mucoid) | 50 | 1340 | 14 | 0 | 0 | 0 |
| CF23 | *P.aeruginosa* (mucoid) | 53 | 47800 | 206 | 0 | 0 | 0 |
| CF42 | *P.aeruginosa* (mucoid) | 48 | 3660 | 1 | 1 | 1 | 2 |
| CF42 | *P.aeruginosa* (mucoid) | 53 | 12040 | 7 | 17 | 4 | 21 |
| CF2 | *S. maltophilia* | 71 | 1200 | 23 | 4 | 22 | 26 |
| CF2 | *S. maltophilia* | 25 | 1500 | 0 | 3 | 0 | 3 |
| CF2 | *S. maltophilia* | 54 | 5000 | 23 | 103 | 23 | 126 |
| CF2 | *S. maltophilia* | 20 | 68000 | 0 | 1 | 0 | 1 |
| CF10 | *S. maltophilia* | 20 | 4620 | 0 | 0 | 0 | 0 |
| CF10 | *S. maltophilia* | 48 | 5000 | 0 | 0 | 0 | 0 |
| CF133 | *S. maltophilia* | 50 | 3100 | 0 | 0 | 0 | 0 |
| CF133 | *S. maltophilia* | 16 | 4080 | 195 | 183 | 81 | 264 |
| CF133 | *S. maltophilia* | 50 | 9000 | 19 | 4 | 2 | 6 |
| CF133 | *S. maltophilia* | 53 | 9560 | 308 | 0 | 0 | 0 |
| CF50 | Burkholderia sp | 50 | 3360 | 0 | 1 | 1 | 2 |
| CF50 | Burkholderia sp | 40 | 8680 | 1 | 0 | 1 | 1 |
| CF50 | Burkholderia sp | 58 | 602000 | 1 | 0 | 0 | 0 |
| CF64 | Burkholderia sp | 39 | 5100 | 1 | 0 | 0 | 0 |
| CF64 | Burkholderia sp | 38 | 1570 | 0 | 0 | 0 | 0 |
| CF66 | Burkholderia sp | 52 | 3336 | 0 | 0 | 0 | 0 |
| CF66 | Burkholderia sp | 22 | 6920 | 0 | 0 | 0 | 0 |

Supplemental Table 1. CF respiratory isolate bioaerosol production during each use of a contaminated nebulizer

| | | | | CFU's recovered post-nebulization | | | |
|---|---|---|---|---|---|---|---|
| **Isolate** | **Bacteria** | **Inoculum (CFU) each nebulizer location** | **Ambient relative humidity (%)** | **100 µl albuterol solution** | **Bioaerosols 3.3-0.98 µM** | **Bioaerosols 14.1-5.39 µM** | **Total recovered from NGI** |
| CF50 | Burkholderia sp | 13650 | 16 | 1 | 12 | 6 | 18 |
| | | | 65 | 50 | 155 | 48 | 203 |
| CF64 | Burkholderia sp | 3080 | 16 | 3 | 1 | 0 | 1 |
| | | | 60 | 25 | 84 | 123 | 207 |
| CF11 | *Pseudomonas aeruginosa* (mucoid) | 5480 | 16 | 18 | 1 | 0 | 1 |
| | | | 65 | 75 | 62 | 118 | 180 |
| CF23 | *Pseudomonas aeruginosa* (mucoid) | 3910 | 20 | 34 | 0 | 0 | 0 |
| | | | 60 | 0 | 8 | 2 | 10 |
| CF9 | *Pseudomonas aeruginosa* (non-mucoid) | 2260 | 22 | 6 | 0 | 0 | 0 |
| | | | 73 | 0 | 0 | 0 | 0 |
| CF17 | *Pseudomonas aeruginosa* (non-mucoid) | 4000 | 23 | 0 | 0 | 0 | 0 |
| | | | 68 | 3 | 178 | 83 | 261 |
| CF56 | *Pseudomonas aeruginosa* (non-mucoid) | 24000 | 20 | 0 | 0 | 0 | 0 |
| | | | 70 | 12 | 0 | 3 | 3 |
| CF1 | *Staphylococcus aureus* | 5660 | 16 | 151 | 761 | 141 | 902 |
| | | | 75 | 164 | 913 | 310 | 1223 |
| CF5 | *Staphylococcus aureus* | 1460 | 16 | 0 | 0 | 0 | 0 |
| | | | 73 | 0 | 0 | 0 | 0 |
| CF7 | *Staphylococcus aureus* | 2580 | 16 | 0 | 0 | 0 | 0 |
| | | | 83 | 218 | 401 | 105 | 506 |
| CF2 | *Stenotrophomonas maltophilia* | 2800 | 16 | 86 | 1 | 0 | 1 |
| | | | 63 | 330 | 715 | 258 | 973 |
| CF10 | *Stenotrophomonas maltophilia* | 1850 | 16 | 7 | 7 | 1 | 8 |
| | | | 60 | 12 | 0 | 8 | 8 |

Supplemental Table 2. Increased relative humidity (RH) increases bioaerosol dispersion after albuterol administration using a contaminated nebulizer. Nebulizers contaminated with the identical inoculum were dried 2 h at either low (≤20%) or high (>60%) RH then used to aerosolize albuterol for bacterial recovery from aerosolized particles.

| | | | | | Recovered CFUs | | | |
|---|---|---|---|---|---|---|---|---|
| **Isolate** | **Bacteria** | **Inoculum (CFU) each nebulizer location** | **Dry time** | **Ambient relative humidity (%)** | **100 μl albuterol solution** | **Bioaerosols 3.3-0.98 μM** | **Bioaerosols 14.1-5.39 μM** | **Total recovered from NGI** |
| CF4 | *S. aureus* | 2350 | 2-h | 64 | 12 | 218 | 66 | 284 |
| | | | 24-h | 72 | 62 | 2 | 14 | 16 |
| CF11 | *P. aeruginosa* (mucoid) | 1350 | 2-h | 75 | 6 | 0 | 4 | 4 |
| | | | 24-h | 72.5 | 0 | 3 | 1 | 4 |
| CF56 | *P. aeruginosa* (non-mucoid) | 5480 | 2-h | 65 | 75 | 2 | 2 | 4 |
| | | | 24-h | 40 | 0 | 1 | 0 | 1 |
| CF2 | *S.maltophilia* | 2430 | 2-h | 64 | 46 | 110 | 81 | 191 |
| | | | 24-h | 72 | 43 | 2 | 1 | 3 |
| CF10 | *S. maltophilia* | 1850 | 2-h | 65 | 12 | 2 | 6 | 8 |
| | | | 24-h | 65 | 4 | 6 | 0 | 6 |
| CF50 | Burkholderia sp | 1240 | 2-h | 56 | 9 | 46 | 28 | 74 |
| | | | 24-h | 63 | 0 | 0 | 0 | 0 |

Supplemental Table 3. Extended drying reduces detachment and bioaerosol dispersion after albuterol administration using a contaminated nebulizer

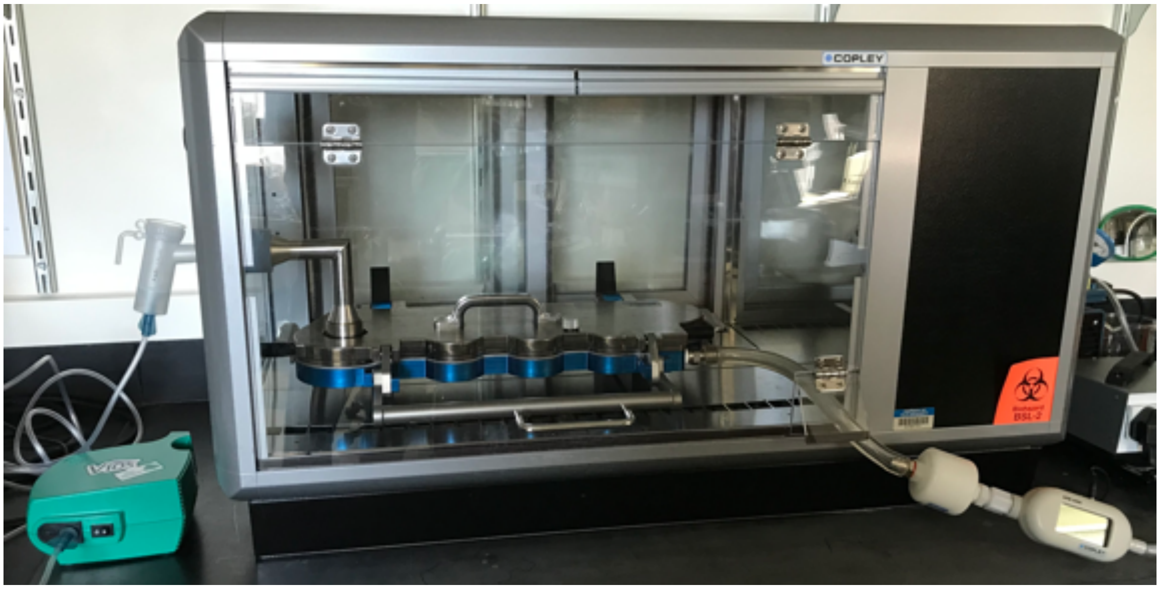


Supplemental Figure 1. Experimental set up for measuring bioaerosols produced during albuterol nebulization with contaminated nebulizers. Albuterol was added to contaminated nebulizers and the bioaerosols of different sizes collected in a refrigerated Next Generation Pharmaceutical Impactor with in-line measurement of the vacuum maintained at 15L/min.

Supplemental References


S1. Berlinski A, Waldrep JC. Four hours of continuous albuterol nebulization. Chest 1998;114(3):847-853.

S2. Berlinski A, Hayden JB. Optimization of a procedure used to measure aerosol characteristics of nebulized solutions using a cooled next generation impactor. J Aerosol Med Pulm Drug Deliv 2010;23(6):397-404.

S3. Joshi PR, Parmar SJ, Patel BA. Spectrophotometric Simultaneous Determination of Salbutamol Sulfate and Ketotifen Fumarate in Combined Tablet Dosage Form by First-Order Derivative Spectroscopy Method. International Journal of Spectroscopy 2013;2013:1-6.

S4. Phanasekar P, Sharma P. DETERMINATION OF ALBUTEROL IN BULK AND DOSAGE FORM BY HPLC USING BUFFER-ETHANOL SYSTEM. Journal of Harmonized Research in Applied Science 2019;7:21.

S5. Merkus PJ, van Essen-Zandvliet EE, Parlevliet E, Borsboom G, Sterk PJ, Kerrebijn KF, et al. Changes of nebulizer output over the years. Eur Respir J 1992;5(4):488-491.

S6. Awad SM, Berlinski A. Crossover Evaluation of Compressors and Nebulizers Typically Used by Cystic Fibrosis Patients. Respir Care 2018;63(3):294-300.

S7. Solomita M, Smaldone GC. Reconciliation of Cascade Impaction during Wet Nebulization. J Aerosol Med Pulm Drug Deliv 2009;22(1):11-18.

S8. Saiman L, Siegel JD, LiPuma JJ, Brown RF, Bryson EA, Chambers MJ, et al. Infection prevention and control guideline for cystic fibrosis: 2013 update. Infect Control Hosp Epidemiol 2014;35 Suppl 1:S1-S67.
